# Visual Scanning and Reading Ability in Normal and Dyslexic Children

**DOI:** 10.1155/2008/564561

**Published:** 2008-04-11

**Authors:** G. Ferretti, S. Mazzotti, D. Brizzolara

**Affiliations:** ^1^Department of Developmental NeuroscienceIRCCS “Stella Maris”PisaItaly; ^2^Division of Child Neurology and PsychiatryUniversity of PisaPisaItaly

**Keywords:** Visual scanning, reading acquisition, developmental dyslexia

## Abstract

Very few studies have investigated the development of visual search of aligned stimuli in relation to normal reading acquisition and in developmental dyslexia.

In this study we used a new computerised experimental task which requires a visuo-motor response (RT) to a target appearing unpredictably in one out of seven different spatial positions on a horizontally aligned array of 18 geometrical figures.

The aims of the study were to investigate: (1) the visual scanning development in normal children from pre-school to school age; (2) whether visual scanning performance in kindergarten children could predict reading acquisition; (3) the visual scanning abilities in a group of developmental dyslexic children.

The main results were: (1) a significant decrement of RTs with age and a progressive increase of the left-to-right gradient with reading experience; (2) visual scanning abilities in kindergarten proved to be a good predictor of reading acquisition; (3) dyslexics were slow scanners and did not present the left-to-right strategy typical of normal readers.

The results support the hypothesis of a relationship between visual scanning and reading abilities.

## 1. Introduction

The role of visual processing in literacy acquisition has been obscured for many years by the prevalent view of reading as a phonologically based process [14,16]. Recently however a new interest for the non linguistic skills involved in reading has produced evidence on the relationship between visual processing and early reading acquisition [10], which may also contribute to the understanding of the cognitive factors underlying developmental dyslexia. Deficits in the spatial distribution of visual attention [7,8], in the visual analysis of "crowded" stimuli [15] and in the visual attention span [17] have been found in developmental dyslexics. An important component of reading, the left-to-right visual scanning, has been however scarcely investigated in normal and dyslexic readers and mainly by means of indirect measures, such as line bisection paradigms [3] or in paper-and-pencils visual search tasks [2,5].

The aim of the present study was to explore the relationship between visual scanning processing and reading acquisition, both in dyslexic and in normal readers, by means of a new computerised paradigm, which requires visual scanning of non alphabetic stimuli.

For this purpose, we collected cross sectional data on visual scanning development from preschool to school age in normal children (experiment 1); by means of longitudinal data we studied the predictive value of visual scanning abilities on reading acquisition (experiment 2); the visual scanning processes were then investigated in a group of developmental dyslexics, with the hypothesis that these children would show an atypical visual scanning performance (experiment 3).

ISSN 0953-4180/08/$17.00 © 2008 – IOS Press and the authors. All rights reserved

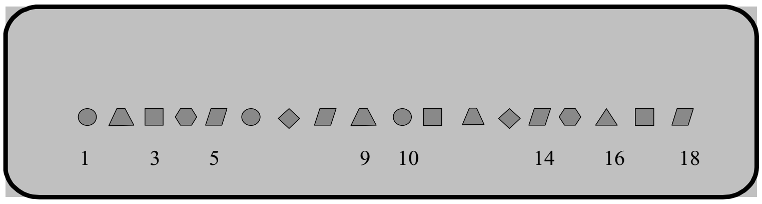

Fig. 1. Experimental task display (VS-18); the target is the triangle.

## 2. EXPERIMENT I

### 2.1. Materials and methods

#### 2.1.1. Subjects

133 right handed children (66 males, 67 females) took part to the experiment: 70 attending kindergarten (mean age: 5y3m ± 7 m), 30 grade 1 (mean age: 6y8m ± 3 m) and 33 grade 3 (mean age: 8y9m ± 3,6 m). Children with diagnosed neurological or psychiatric disorders were excluded.

#### 2.1.2. Tests and procedure

In the Visual Scanning test (VS-18) an horizontally aligned array of 18 geometric figures, subtending 1°, were presented on a PC screen. The child was asked to respond, pressing the space-bar, as soon as he/she detected the target (triangle), randomly appearing in the positions 1, 3 and 5 on the left, 14, 16 and 18 on the right and alternatively in the central positions 9 or 10 (Fig. 1). Immediately after the motor response, subjects were asked to point to the target. The test consisted of 35 trials (5 for each target position), which were preceded by an acoustic warning signal. Mean Reaction Times (RTs) for each target position were collected. Before the experimental session the children underwent a short training.

We hypothesised that in normal readers a typical left-to-right scanning strategy would result in progressively slower Reaction Times (RTs) from P1 to P18.

For statistical analysis the following values were calculated: mean RT for each target position, mean RT for all targets (Tot-RT) and a regression coefficient b, as index of the slope of the regression line.

### 2.2. Results

Mean RTs for the different target positions in the three age groups are visualized in Fig. 2.

An ANOVA with Age (3 levels) and Gender (2 levels) as unrepeated factors and target Position (7 levels) as repeated factor showed significant Age ($F_{(2,127)} = 33,411$ $p < 0.0001$) and Position ($F_{(6,762)} = 22,08$ $p < 0.0001$) effects and a significant Age x Position interaction ($F_{(12,762)} = 1,783$ $p = 0.047$).

Neither gender effect nor interactions were significant.

Seven one-way ANOVA on the RTs of each position yielded a significant main effect of Age ($p < 0.0001$). A post-hoc analysis (Tukey) indicated that all comparisons were significant in the three left positions (P1, P3 and P5), while for the three right positions (P14, P16 and P18) they were significant only between kindergarten and grade 3; between grade 1 and 3 a significant difference was found only in P18.

A similar one-way ANOVA, carried on regression coefficient b, showed a significant Age effect ($F_{(2,132)} = 4,104$ $p = 0.019$). Post-hoc comparisons (Tukey) were significant between pre-readers (kindergarten) and grade 3 children.

The experiment showed that the RTs for the seven target positions changed with age, with increasingly faster RTs to targets in the left positions than in the right. The idea of a progressive development of the left-to-right scanning strategy from pre-school to school age is also supported by the change with age in the slope of the regression line.

Highly significant correlations between the mean Tot-RT and reading speed ($r = -0,424$ $p < 0.001$) and accuracy ($r = -0,435$ $p < 0.0001$), expressed in z scores, were found in the school age children, supporting the hypothesis of a relation between reading performance and visual processing efficiency.

## 3. EXPERIMENT 2

### 3.1. Materials and methods

#### 3.1.1. Subjects, tests and procedure

The sub-sample of 32 children attending the last year in kindergarten was followed-up and assessed at the end of grade 1 by means of the MT test [4], which measures reading speed and accuracy of short passages.

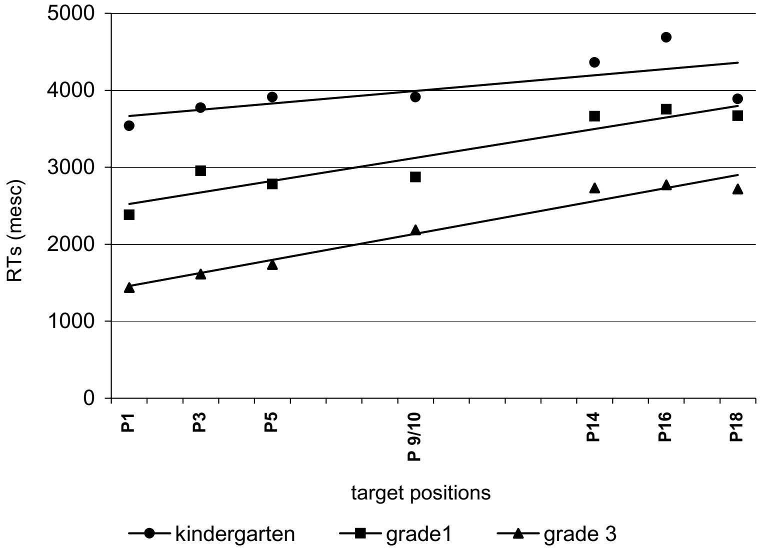

Fig. 2. Reaction Times to the seven target positions in the VS-18 across ages.

Number of errors and reading speed were scored and then converted to z scores according to the normative data provided for the different school levels.

### 3.2. Results

Significant correlations were found between the mean Tot-RT in VS-18 measured in kindergarten and reading speed ($r = -0,412$ $p < 0.05$) and accuracy ($r = -0,35$ $p < 0.05$), expressed in z scores, assessed the year after, when the same children were in grade 1.

An ANOVA on speed and accuracy reading scores of children classified as slower and faster visual scanners in kindergarten (Tot-RT above or below the $50^{th}$ percentile) showed a trend for a significant difference for accuracy ($F_{(1,31)} = 3,493$ $p = 0.07$). When ranking the children in four quartiles on the basis of their RTs, reading speed and accuracy differed significantly (respectively $F_{(3,31)} = 4,782$ $p = 0.008$ and $F_{(3,31)} = 4,181$ $p = 0.014$). Post-hoc analysis showed that reading speed of children in the fourth quartile ($n = 8$) differed significantly from that of children of the other three quartiles. In terms of reading accuracy, children in the fourth quartile differed from those of the second and third quartiles (see Fig. 3).

Reading speed and accuracy were correlated either in the total sample ($n = 32$, $r = 0.879$ $p < 0.001$) or in the second, third and fourth quartiles (while there was a trend in the first).

The visual scanning speed in kindergarten was found to be a significant predictor of reading abilities: the faster the visual scanning performance in pre-schoolers, the more efficient the decoding skill at the end of the first grade.

## 4. EXPERIMENT 3

### 4.1. Materials and methods

#### 4.1.1. Subjects

20 dyslexic children, 15 males and 5 females (mean age: 9y4m, range 8y5m–10y9m) were selected from a sample of children referred for learning difficulties to the Department of Developmental Neuroscience of the Stella Maris Scientific Institute. The diagnosis of developmental dyslexia was made according to the DSM-IV criteria: intelligence within normal limits (z score>-1 SD), impaired scores on standardised reading tests (z score<-2 SD for either speed or accuracy in at least one of the reading tests), no neurological and/or sensory deficit or emotional disturbance and regular school attendance. One male subject had been previously diagnosed as ADHD, according to DSM-14 criteria.

A control group of 20 children was matched for gender and age.

#### 4.1.2. Tests and Procedure

Intelligence level was assessed by Raven's Coloured Progressive Matrices, according to Italian normative data [11]; reading speed and accuracy of single words and non-words was assessed by means of subtests 4 and

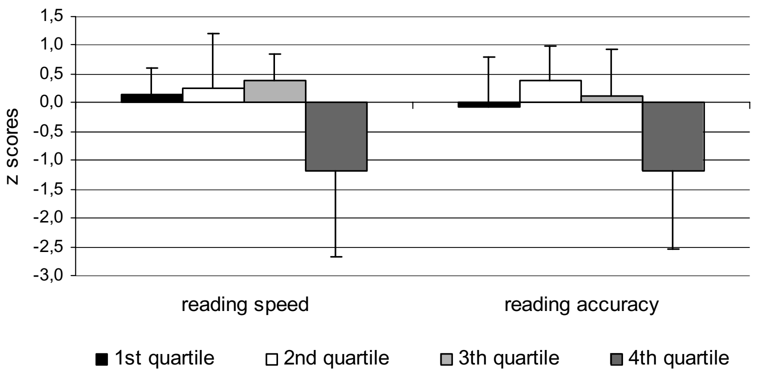

Fig. 3. Reading speed and accuracy in grade 1 of children ranked in four quartiles on the basis of their visual scanning RTs assessed in kindergarten.

5 of Sartori, Job & Tressoldi's Developmental Dyslexia and Dysgraphia Battery [12], which provides normative data (means, SDs) from second to eighth grade.

Visual scanning was assessed with the VS-18 software (described in Exp.1).

### *4.2. Results*

Mean RTs for the different target positions in the two groups are visualised in Fig. 4.

An ANOVA with Group (2 levels) as unrepeated factor and target Position (7 levels) as repeated factor showed highly significant Position ($F_{(6,228)=}$27,509 $p < 0.0001$) and Group ($F_{(1,38)=}$4,457 $p = 0.041$) effects and a significant Group x Position interaction ($F_{(6,228)} =$ 5,399 $p < 0.0001$). Seven one-way ANOVA on the RTs of each position indicated a significant main effect of Group for P1 ($F_{(1,39)} = 17,517$ $p < 0.0001$), P3 ($F_{(1,39)} = 18,619$ $p < 0.0001$) and P5 ($F_{(1,39)} = 11,960$ $p = 0.001$), being dyslexics slower on these three left positions. On the contrary, no significant differences between dyslexics and controls were found on the right side.

A one-way ANOVA on the regression coefficient b demonstrated a significant Group effect ($F_{(1,39)} = 10,815$ $p = 0.002$), with dyslexics showing an almost flat regression line on the seven target positions, while in the control group a linear RTs increase from left-to-right was observed (see Fig. 4).

Dyslexic children, according to our hypothesis, proved to be poor scanners: their RTs did not linearly increase from left-to-right as in normal readers: while the latter started their visual search from the left side of the array, with faster RTs for these positions, dyslexics did not show this visual search strategy and therefore their RTs did not differ from left to right.

## 5. Discussion

Our data show a clear relationship between visual scanning and reading abilities. Two sets of data support this conclusion: 1) speed of processing in a visual search task in kindergarten children was found to be a good predictor of early literacy acquisition, which on turn influences the development of a left-to-right visual scanning strategy; 2) dyslexics were less efficient both in speed of processing and in left-to-right scanning strategy, compared to age matched controls.

Cross-sectional data in children from pre-school to school age showed a significant decrease of RTs and the development of a more regular left-to-right scanning strategy which favours stimuli located on the left side of the array. Since it has been demonstrated that visual processing speed in kindergarten predicts reading performances at the end of grade 1, and RTs linear increase from left to right develops after schooling, it may be assumed that visual processing efficiency and left-to-right scanning are differently linked to reading acquisition, the first influencing, the second being influenced by reading practice.

As expected, dyslexics, unlike normal readers, showed a slower and atypical visual search performance. The fact that dyslexics did not show differences in RTs between left and right target positions may be due to a random visual access to the array, which on consequence does not advantage the first positions of the alignment. Deficits in visual search tasks have been already found in poor readers and dyslexics [2,5]; by means of our paradigm we could measure the speed of the response as a function of the position of the stimulus along the array, thus obtaining a more direct tracking index of the scanning procedure.

The lack of the left-to-right scanning strategy found in dyslexics may be influenced by the reduced reading

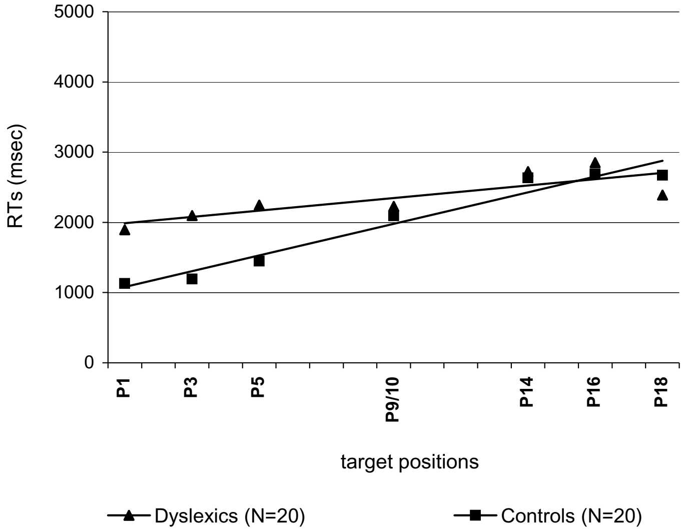

Fig. 4. RTs in the VS-18 in dyslexics and controls.

practice, but also may be due to visual attention abnormalities. Developmental dyslexics have been found by Facoetti et al. [6] to be impaired in tasks requiring pre-orienting and focusing automatic attention toward regions of visual space where stimuli are expected to appear. Another attention explanation of dyslexia has been recently put forward, according to which dyslexic children might be affected by a kind of "mini neglect" [8,9,13]. Moreover, a visual attention span deficit, which consists in a reduced efficiency in processing of visual aligned stimuli, has been found to impair reading performance, even in absence of phonological difficulties [1].

Although reading disabilities have been most frequently explained on the basis of phonological deficit, faulty mechanisms in the visual analysis and selection of orthographic units may play a role in developmental dyslexia, contributing to the difficulty in establishing analytic grapheme-phoneme sequential mappings.

## Acknowledgements

This study is part of the PhD research project of the second author (S. Mazzotti).

We thank Dr. Anna Chilosi for referring us some of the dyslexic children and Filippo Gasperini, who collected some reading data.
